# Optimization of Nanofiltration Hollow Fiber Membrane Fabrication Process Based on Response Surface Method

**DOI:** 10.3390/membranes12040374

**Published:** 2022-03-29

**Authors:** Mingshu Wang, Chang Liu, Min Fan, Meiling Liu, Songtao Shen

**Affiliations:** 1 School of Environment and Resources, Southwest University of Science and Technology, Mianyang 621000, China; wangms0826@163.com (M.W.); firstfanmin@163.com (M.F.); xnkdlml@163.com (M.L.); shensongtao@swust.edu.cn (S.S.); 2 Low Cost Wastewater Treatment Technology International Science and Technology Cooperation Base of Sichuan Province, Mianyang 621000, China

**Keywords:** layer-by-layer self-assembly, multiple regression analysis, nanofiltration membrane, process optimization, response surface methodology

## Abstract

Layer-by-layer (LBL) self-assembly technology has become a new research hotspot in the fabrication of nanofiltration membranes in recent years. However, there is a lack of a systematic approach for the assessment of influencing factors during the membrane fabrication process. In this study, the process optimization of LBL deposition was performed by a two-step statistical method. The multiple linear regression was performed on the results of single-factor experiments to determine the major influencing factors on membrane performance, including the concentration of Poly (allylamine hydrochloride) (PAH), glutaraldehyde, and the NaCl concentration in PAH solution. The Box–Behnken response surface method was then used to analyze the interactions between the selected factors, while their correlation with the membrane performance was obtained by polynomial fitting. The R^2^ value of the regression models (0.97 and 0.94) was in good agreement with the adjusted R^2^ value (0.93 and 0.86), indicating that the quadratic response models were adequate enough to predict the membrane performance. The optimal process parameters were finally determined through dual-response surface analysis to achieve both high membrane permeability of 14.3 LMH·MPa^−1^ and MgSO_4_ rejection rate of 90.22%.

## 1. Introduction

Nanofiltration (NF) is a pressure-driven process between reverse osmosis and ultrafiltration. The pore size of the NF membrane is about 1~2 nm, with a molecular weight cut-off (MWCO) between 100~5000 Da. It can effectively remove hardness and other micro-pollutants from wastewater and thus is widely used in drinking water treatment, seawater/brackish water desalination and advanced treatment for sewage reuse [1,2,3]. At present, the fabrication methods for NF membranes mainly include phase inversion, surface coating/grafting, interfacial polymerization and layer-by-layer (LBL) self-assembly [4,5]. Among them, LBL technology has become a new research hotspot due to its simple operation, environmental friendliness and low preparation cost [6,7]. First of all, LBL can be simply carried out on essentially any membrane substrate that supports the adsorption of the initial layer of the polymer, and enables its universal and convenient surface chemistry tailoring [8]. Secondly, the flexibility and versatility of the LBL process has made it possible to design specific membranes for targeted applications such as pervaporation, decontamination, resource recovery and so on [9,10,11]. In general, a composite selective layer can be formed upon alternative deposition of polyelectrolytes with opposite charges on the substrate membrane surface. Various studies have proven that the performance of LBL NF membranes is greatly affected by the deposition conditions, such as the number of deposited layers, the concentration of the polyelectrolyte solution and the ionic strength of the respective solution [12,13,14]. However, those studies were only limited to the trial-and-error methods focusing on the influence of a single process parameter, while the simultaneous influence of various factors and their interactions were seldomly explored [15,16]. Therefore, a more systematic approach for the assessment of influencing factors during the LBL membrane fabrication process is needed.

Statistical optimization methodologies such as orthogonal tests and neural network models are widely used to determine the optimal parameters of membrane fabrication processes [17,18]. Among all of these, response surface methodology (RSM) can model and analyze the conditions in which a target parameter (response) is influenced by multiple variables by evaluating their relative significance, even in the presence of complex interactions, while displaying the results using visual graphics and digital technology [19,20]. RSM is thus adopted to study the contribution of membrane fabrication/operational conditions and their inherent interactions towards the maximization of membrane performance with reduced number of experiments and higher test precision [21,22]. For instance, M. Khayet et al. [23] used RSM to optimize the UV irradiation intensity/duration, monomer concentration and other factors of the UV-initiated graft-polymerization process, and established a prediction model for the resulting NF membrane performance. Wang et al. [24] optimized various fabrication parameters such as the sodium alginate (SA) content, electric voltage, receiving distance and the nozzle velocity during electrospinning process via RSM and revealed that the SA content had a major effect on the diameter of the fibers.

In this study, a two-step statistical approach was adopted to investigate the influence of various parameters on the fabrication process of LBL nanofiltration membranes. Firstly, single-factor experiments were conducted to determine the major influencing factors on the membrane performance through multiple linear regression (MLR) analysis. The optimum fabrication conditions and their interrelationships were then determined by Box–Behnken design (BBD) experiments established by RSM. The validity of the quadratic response model was tested with analysis of variance (ANOVA) and evaluated by comparing the observed membrane performance with the predicted results from the model.

## 2. Materials and Methods

### 2.1. Materials and Chemicals

Ultrafiltration (UF) hollow fiber membranes (MWCO = 30,000, inner diameter = 1.0 mm) were purchased from Shandong Jinhui Membrane Technology, China as substrate. Polystyrene sodium sulfonic acid salt (PSS, Mw = 500 kDa, Alfa Aesar, Shanghai, China), poly (allylamine hydrochloride) (PAH, Mw = 120–200 kDa, Alfa Aesar, Shanghai, China) and glutaraldehyde (GA, 50% in water, Maclin, Shanghai, China) were used for LBL deposition process. Sodium chloride (NaCl, Kelon Chemical Reagent, Chengdu, China), magnesium chloride hexahydrate (MgCl_2_·6H_2_O, Kelon Chemical Reagent, Chengdu, China), sodium sulfate (Na_2_SO_4_, Aladdin Biochemical, Shanghai, China) and magnesium sulfate heptahydrate (MgSO_4_·7H_2_O, Aladdin Biochemical, Shanghai, China) were used for NF performance tests. Deionized (DI) water was produced by an ultrapure water machine (Youpu, Chengdu, China).

### 2.2. Preparation of NF Membrane

**Figure 1:**
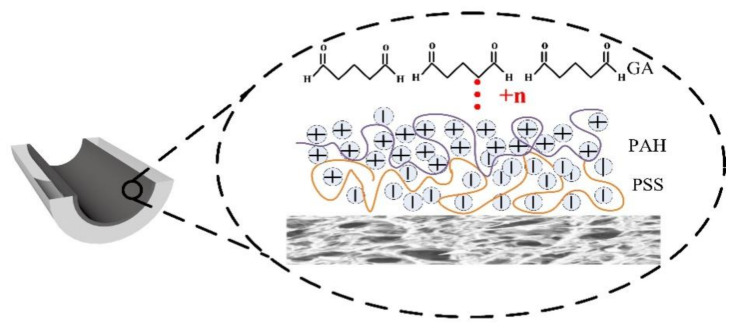
Schematic drawing of NF hollow fiber membrane prepared by LBL method.

The dried hollow fibers were sealed into plastic membrane modules with an effective length of 25 cm. LBL deposition at the inner surface of the substrate was performed by introducing the polyelectrolyte solution throughout the fiber lumen with a syringe and maintained for 5 min. Polyanion PSS and polycation PAH solutions were applied alternately to achieve the desired number of layers with a 5 min DI water rinse in between. GA crosslinking was conducted afterwards in a similar fashion, if applicable. The schematic drawing of the deposition process is shown in Figure 1. The membrane modules were then stored in DI water for NF performance tests.

### 2.3. Membrane Performance Test

**Figure 2:**
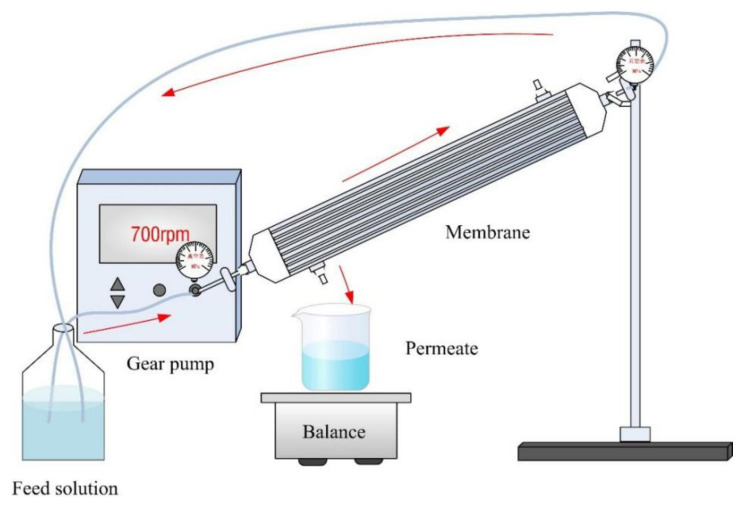
The cross-flow filtration device.

A cross-flow filtration set-up (as shown in Figure 2) was used to investigate the pure-water permeability of NF membranes and their rejection rate towards four salt solutions (NaCl, Na_2_SO_4_, MgCl_2_ and MgSO_4_, 1 g·L^−1^, respectively) based on the conductivity measurements (DDBJ-351L, LeiCi, Shanghai, China). The feed solutions were passed through the membrane lumen under a pressure of 0.2 MPa and the results were measured after 30 min for stabilization. Three parallel experiments were conducted and the average results were reported. The pure-water permeability (*J,* LMH·MPa^−1^) was calculated by Equation (1):

(1) $$J=\frac{V}{A\Delta t\Delta p}$$

where *V* is the volume of the permeate, L; Δp is the transmembrane pressure, MPa; *A* is the effective area of the membrane, m^2^; Δt is sampling time, h.

The salt-rejection rate (*R*) was calculated by Equation (2).

(2) $$R=\frac{C_{F}-{\;C}_{P}}{C_{F}}\;\times 100\%$$

where $C_{F}$ is the mass concentration of permeate, g·L^−1^; $C_{P}$ is the mass concentration of salt solutions, g·L^−1^.

### 2.4. Single-Factor Experiments

A total of 30 groups of salt-rejection experiments (independent variable values were selected based on our previous studies and are shown in Table 1 [25,26,27], including PSS concentration (X_1_, g·L^−1^), NaCl concentration in PSS solution (NaCl_PSS_, X_2_, mol·L^−1^), PAH concentration (X_3_, g·L^−1^), NaCl concentration in PAH solution (NaCl_PAH_, X_4_, mol·L^−1^), number of layers (X_5_) and GA concentration (X_6_, %)) were conducted to evaluate the NF membrane performance (membrane permeability and rejection rate of four salt solutions). Based on the preliminary results, the other variables were set as follows: number of layers was 2; PSS concentration was 5 g·L^−1^; NaCl_PSS_ was 0.5 mol·L^−1^; PAH concentration was 5 g·L^−1^; NaCl_PAH_ was 0.5 mol·L^−1^; and GA concentration was 0 g·L^−1^. Statistical Product and Service Solutions (SPSS) software was then used for MLR analysis. Student’s *t*-test was adopted to determine three variables with the highest correlation with the membrane performance for the following response surface experiments.

### 2.5. Response Surface Experiments

A Box–Behnken design (BBD) for the response surface methodology (RSM) study was then used to optimize the LBL deposition conditions for membrane performance enhancement, where membrane permeability and MgSO_4_ rejection were chosen as the response variables. Based on the single-factor experiment results, PAH concentration (X_3_, g·L^−1^), NaCl_PAH_ (X_4_, mol·L^−1^), and GA concentration (X_6_, %) were selected as the independent variables with their actual levels and corresponding codes listed in Table 2. The other variables were set as follows: X_1_ = 5.00 g·L^−1^, X_2_ = 0.50 mol·L^−1^, X_5_ = 2.5 layers. The BBD generated by Design-Expert statistical software combined three factors at three levels, namely high (+1), low (−1), and center (basic level; 0). The center points were the intermediate values between the high and low levels. Basic-level experiments were repeated to ensure the model stability [28]. Then, 17 sets of deposition combinations including 3 sets of center points with five replications were adopted in this study where the number of experimental runs was determined by Equation (3) [29]

(3) $${N=k}^{2}{+k+c}_{p}$$

where *k* is the factor number and *c_p_* is the replicate number of the central point.

Second-order polynomials were employed to fit the BBD experimental data while a quadratic model was generated from the data according to the following equation:

(4) $${y=\beta }_{0}+\overset{K}{\underset{i=1}{\sum }}\beta_{i}x_{i}+\overset{K}{\underset{i=1}{\sum }}\beta_{ii}x_{i}+\overset{K}{\underset{i\text{<}1}{\sum }}\beta_{ij}x_{i}x_{j}+\varepsilon$$

where *y* is the predicted response (membrane permeability/MgSO_4_ rejection rate); *β*_0_, *β_i_*, *β_ii_*, and *β_ij_* represent the regression coefficients for the term intercept and the linear, square, and interaction effects, respectively; *x_i_* and *x_j_* refer to the coded levels of the design variables.

The least-square estimations of the regression coefficients were computed using the multiple regression method [30]. The model adequacy was tested with analysis of variance (ANOVA), where statistical estimators such as square of regression (R^2^), adjusted square of regression (R_adj_^2^), Fisher value (F-value) and probability (*p*-value) were applied to measure the statistical significance of the model and the variables.

## 3. Results and Discussion

### 3.1. Single-Factor Experiments

#### 3.1.1. Salt-Rejection Experiment

**Figure 3:**
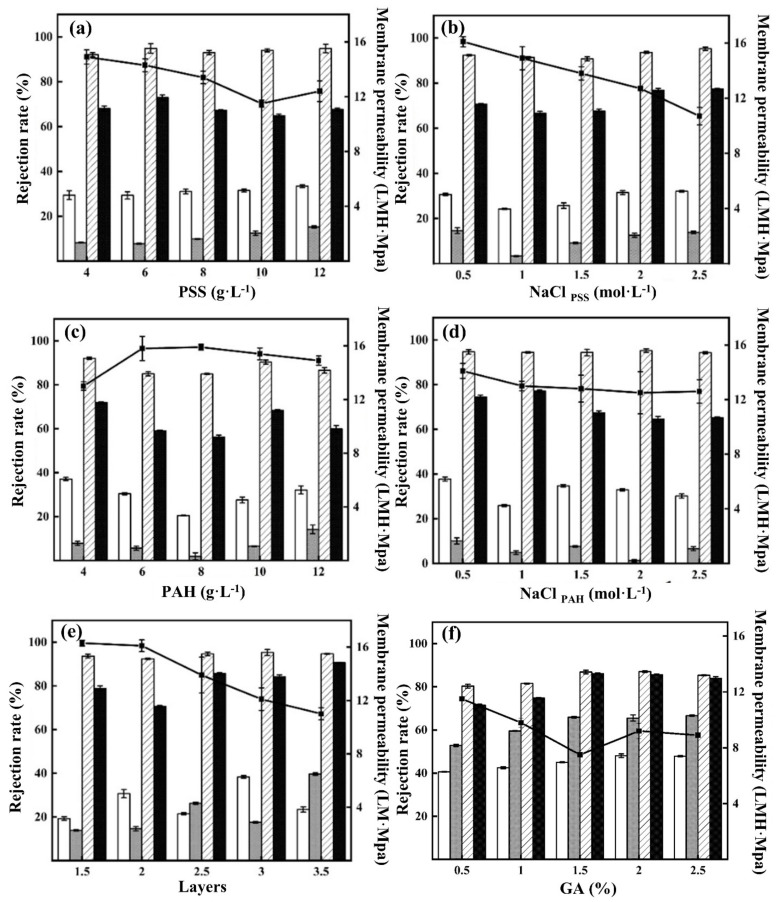
Effects of variables (**a**): PSS; (**b**): NaCl_PSS_; (**c**): PAH; (**d**): NaCl_PAH_; (**e**): layers; (**f**): GA on the performance of LBL NF membranes. (Membrane permeability 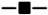, NaCl 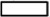, Na_2_SO_4_ 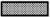, MgCl_2_ 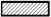, MgSO_4_ 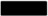).

As can be seen from Figure 3a–e, the rejection against four representative salt solutions for all uncrosslinked membranes are in the order of MgCl_2_ > MgSO_4_ > NaCl > Na_2_SO_4,_ which is in line with our previous studies [25]. This is because an excess amount of PAH monomers is usually accumulated within the typical PSS/PAH multilayers due to their high charge density and relatively small monomer size, resulting in a positive surface charge [25]. Moreover, the high MgCl_2_ rejection (over 80%) observed in all cases indicated no pinholes were formed in the membrane-selective layers, and thus the chosen ranges of independent variables were reasonable. During the chemical crosslinking, the primary amines in PAH react with aldehyde groups of the GA molecule to form the Schiff base (-CQN-) to reduce the molecular weight cut-off as well as the surface charge of the membrane [31]. Therefore, the MgCl_2_ rejections are reduced due to the weakened charge repulsion after crosslinking, as shown in Figure 3f. In contrast, the rejections for the other salts are increased with the enhanced size exclusion, while the permeability decreases following the permeability-selectivity trade off. However, no clear trend for the purpose of membrane performance optimization could be directly obtained from the salt-rejection experiments. Therefore, a two-step statistical approach was then conducted to analyze the results obtained from the rejection tests. The influence of various parameters on the fabrication process of LBL nanofiltration membranes was also investigated to determine the optimum conditions.

#### 3.1.2. Multiple Linear Regression Analysis

Results derived from the MLR analysis are shown in Table 3 (the rejection rate of MgSO_4_ was chosen as the major evaluation criteria here as the general performance indicator for the commercial nanofiltration membrane) and Tables S1–S4 to rank the significance of variables X_1__–6_ on the rejection rate of four salt solutions and membrane permeability. The significance of coefficients was judged by the Student’s *t*-test, which revealed that *p*-values for terms associated with X_3_ (PAH concentration), X_5_ (deposition layer) and X_6_ (GA concentration) can be considered to exert a significant impact on MgSO_4_ rejection rate (*p* < 0.05 means significant) [32]. However, only 0.5 layer (either one PSS or PAH layer) variation could be manipulated for the variable X_5_, and thus it was eliminated as the discrete variable. In the consideration of the absolute values of coefficients and the *p*-value for the remaining variables [33,34], NaCl_PAH_ (X_4_) was then substituted in to ensure the compatibility for the following RSM study.

### 3.2. Response Surface Experiments

#### 3.2.1. RSM Modelling and ANOVA Analysis

The design and results of the three-factor response surface experiment for MgSO_4_ rejection and membrane permeability (to ensure the practical permeability can be achieved simultaneously) are shown in Table S5. Design-Expert software in RSM environment was used to analyze the data and the quadratic response models for MgSO_4_ rejection (Y_1_)/membrane permeability (Y_2_) were obtained as Equations (5) and (6):

(5) $$Y_{1}=+88.24\;-\;5.52*X_{3}+3.03*X_{4}+3.42*X_{6}+3.49*X_{3}*X_{4}+2.45*X_{3}*X_{6}+2.49\;*X_{4}X_{6}\;-\;8.91{*X_{3}}^{2}$$

(6) $$Y_{2}=+9.75+0.56*X_{3}\;-\;1.38*X_{4}\;-\;1.28*X_{6}\;-\;0.9*X_{4}*X_{6}+1.10{*X_{3}}^{2}\;-\;0.72{*X_{6}}^{2}$$

where X_3_, X_4_, and X_6_ are the coded values for the three variables, i.e., PAH concentration, NaCl_PAH_ and GA concentration, respectively.

**Figure 4:**
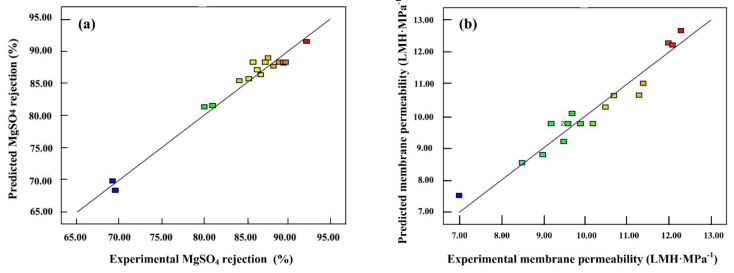
Predicted MgSO_4_ rejection rate (**a**) and membrane permeability (**b**) versus their corresponding experimental measurements.

To ensure the accuracy of the model, a model significance test was performed by applying ANOVA, and the results are shown in Table 4 and Table 5. For significance at the 95% confidence level, factors with *p*-value < 0.05 are considered to be statistically significant [35,36]. According to the test, the *p*-value for both models were less than 0.01, indicating their high significance. Meanwhile, the *p*-values of lack-of-fit (0.40 and 0.14) were not significant relative to the pure error. It revealed that both models could be accepted from a statistical point of view for the prediction of the response in the considered range of variables. The goodness-of-fit for both models were also tested using the determination coefficient R^2^ as shown in Figure 4. It can be seen that the experimental values of the design fit well with the predicted values, where the R^2^ value of the regression models (0.97 and 0.94) was in good agreement with the adjusted R^2^ value (0.93 and 0.86), indicating the quadratic response models were adequate enough to predict the membrane performance.

#### 3.2.2. Effect of LBL Deposition Condition on Membrane Performance by RSM Analysis

According to Table 4, the order of significant factors in the MgSO_4_ rejection response model is as follows: X_3_ ≈ X_3_^2^ < X_6_ < X_4_ < X_3_X_4_ < X_3_X_6_ < X_4_X_6_. The lowest *p*-value of X_3_ and X_3_^2^ are less than 0.0001 among all factors, showing that the PAH concentration has the most prominent effect on membrane rejection. However, the negative sign of both coefficients in Equation (4) indicates that the increment of PAH concentration will decrease MgSO_4_ rejection, which is in correspondence with the single-factor experiment results. In addition, all interaction variables (X_3_X_4_, X_3_X_6_, X_4_X_6_) in this model have *p*-values of less than 0.05, revealing the existence of interaction effects for all variable combinations which will be discussed later on. In contrast, the significant factors of membrane permeability are in the order of: X_4_ ≈ X_6_ < X_3_^2^ < X_3_ < X_4_X_6_ < X_6_^2^, as shown in Table 5. The NaCl concentration in PAH solution (X_4_) and the GA concentration (X_6_) are the key factors determining the thickness and the pore size of the selective layer, and therefore have greater influence on the membrane permeability [25,27]. The only noticeable interaction effect is also observed between those two variables in this case.

**Figure 5:**
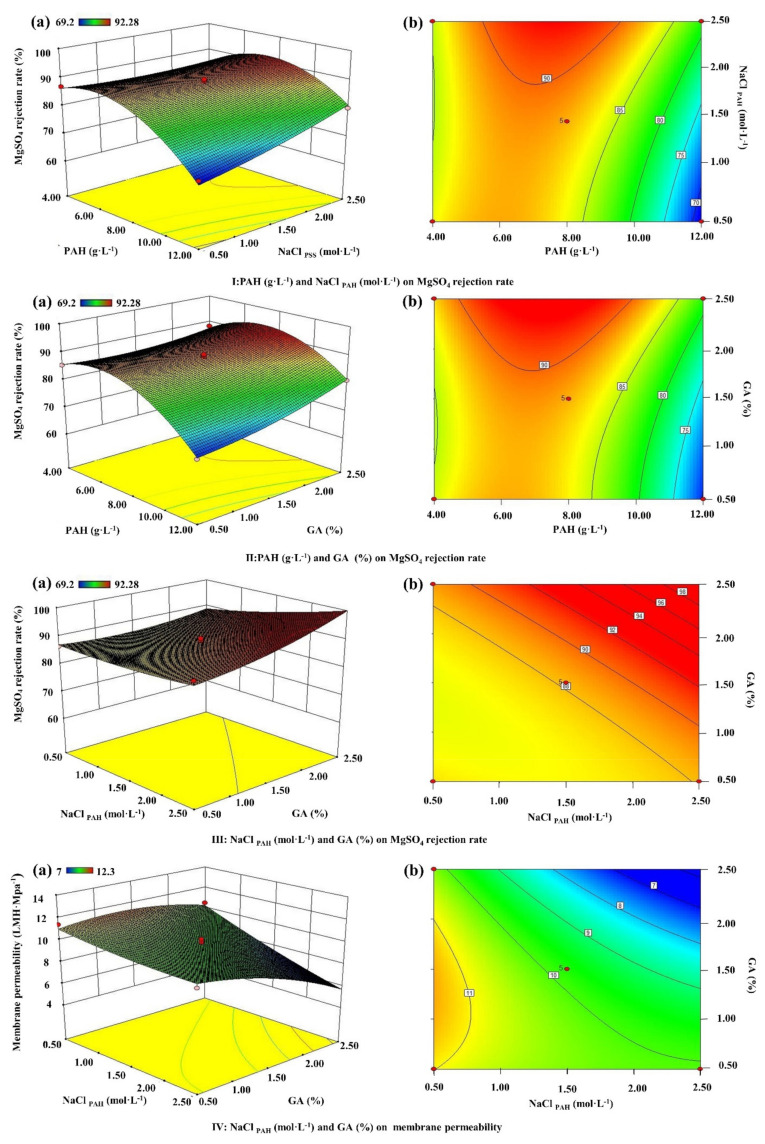
Response surface plots (**a**) and contour maps (**b**) of MgSO_4_ rejection (**I**–**III**) and membrane permeability (**IV**) influenced by the interaction of different factors.

Three-dimensional response surface plots and contour line maps of MgSO_4_ rejection rate (Figure 5I–III) and membrane permeability (Figure 5IV) were obtained from the Design-Expert software for the visualization of the predicted models in RSM. The response surface plot is a theoretical three-dimensional plot showing the relationship between independent variables and the response when changing any two of the variables, while the third was adjusted at the central point [37]. The bottom of the response surface plot is the contour map where lines of the constant response are drawn in the plane of the independent variables. An analysis was then conducted to find out the optimum value of each variable yielding the maximum response, and to understand their interaction effects on the response.

The interaction effect of PAH concentration (X_3_) and NaCl_PAH_ (X_4_) on MgSO_4_ rejection is shown in Figure 5I, while the GA concentration (X_6_) was maintained at the center level of 1.5%. In principle, a more stoichiometric multilayer, determined by the charge density, molar ratio of poly-cations and poly-anions as well as the ionic strength of the solutions, will lead to a better ion pairing among polyelectrolyte chains to form denser selective layer [38]. Therefore, the maximum output zone of MgSO_4_ rejection (>90%) occurs near the upper boundary of the counter line map in Figure 5I, where PAH concentration ranges from 5.5 to 9.5 g·L^−1^ while the NaCl_PAH_ is about 1.8–2.5 mol·L^−1^ for a fixed PSS solution composition (PSS concentration of 5.00 g·L^−1^, NaCl_PSS_ of 0.50 mol·L^−1^). The increment of ionic strength of PAH solution (by the addition of supporting electrolyte NaCl) enhanced the charge screening along the PAH chains to form more coiled and loopy structures [34]. Therefore, more PAH monomers could be adsorbed during the deposition process, and consequently, the thickness of the individual layer will increase with a better MgSO_4_ rejection [32]. In contrast, the further increment of PAH concentration will lead to more extrinsic charge compensation (ion pairing with charged counter-ions in the solution) on the membrane surface, leading to a less tight multilayer structure for lower salt rejection [39].

The crosslinking agent GA can effectively limit the swelling of the LbL layers by reacting with the amine groups in PAH to form a 3D crosslinked polyelectrolyte network. Therefore, the range of PAH concentration within the maximum output zone could be further extended by 26% at higher concentrations of GA (>2%), as shown in Figure 5II. In addition, the synergistic effect between NaCl_PAH_ and GA can be clearly observed from Figure 5III, where the increment of both concentrations will result in better MgSO_4_ rejection up to 98%. However, such a high inter-chain network density would obviously increase the resistance against water permeating through the selective layer, which results in the deduction of water permeability to less than 7 LMH·MPa^−1^ when NaCl_PAH_ and GA are at the highest values of 2.50 mol·L^−1^ and 2.5%, respectively, as seen in Figure 5IV.

#### 3.2.3. Optimization of the LBL Deposition Process

According to Equation (5), the optimal deposition conditions could be determined as a PAH concentration of 5.79 g·L^−1^ with 1.98 mol·L^−1^ NaCl and GA solution concentration of 2.43%. The resulting MgSO_4_ rejection rate was 94.20% (93.89% in prediction) with a membrane permeability of 5.95 LMH·MPa^−1^. Although a high MgSO_4_ rejection rate could be obtained, the relatively low membrane permeability was not ideal for the practical application. Therefore, dual-response surface analysis was adopted by solving Equations (5) and (6) jointly, to realize the simultaneous optimization of MgSO_4_ rejection rate and membrane permeability. The resulting optimal deposition conditions were 5.43 g·L^−1^ PAH concentration with 0.5 mol·L^−1^ NaCl and 0.5% GA concentration. The corresponding MgSO_4_ rejection rate and membrane permeability were 90.22% (90.73% in prediction) and 14.30 LMH·MPa^−1^ (11.20 LMH·MPa^−1^ in prediction), respectively.

## 4. Conclusions

Process optimization of LBL nanofiltration membrane fabrication was conducted in this study to enhance the membrane performance by statistically designed experiments. The most significant parameters determined through MLR analysis of single-factor experiments were the concentration of PAH and GA and the NaCl concentration in PAH solution. The quadratic response model established by the BBD experiments clarified the mathematical relationship between the deposition conditions and the membrane performance, while ANOVA demonstrated the high fitness and feasibility of the models. The optimal deposition conditions were realized by RSM as 5.43 g·L^−1^ of PAH with 0.50 mol·L^−1^ NaCl and 0.50% GA concentration for a MgSO_4_ retention rate of 90.22% and water permeability of 14.30 LMH·MPa^−1^. Overall, it can be concluded that the combination of MLR analysis and RSM is a systematic and scientific approach for the process optimization of membrane fabrication.

## Figures and Tables

**Table 1:** Independent variable values for single-factor experiments.

Variables	Symbols	Values				
PSS (g·L^−1^)	X_1_	4.00	6.00	8.00	10.00	12.00
NaCl_PSS_ (mol·L^−1^)	X_2_	0.50	1.00	1.50	2.00	2.50
PAH (g·L^−1^)	X_3_	4.00	6.00	8.00	10.00	12.00
NaCl_PAH_ (mol·L^−1^)	X_4_	0.50	1.00	1.50	2.00	2.50
Layers	X_5_	1.50	2.00	2.50	3.00	3.50
GA (%)	X_6_	0.50	1.00	1.50	2.00	2.50

**Table 2:** Independent variables and their coded levels for RSM design.

Variables	Symbols	Actual Values of Coded Levels		
		−1.00	0.00	1.00
PAH (g·L^−1^)	X_3_	4.00	8.00	12.00
NaCl_PAH_ (mol·L^−1^)	X_4_	0.50	1.50	2.50
GA (%)	X_6_	0.50	1.50	2.50

**Table 3:** Regression model results of MgSO_4_ rejection rate.

Predictor	Coefficient	Standard Error	t-Value	*p*-Value *
X_1_	−0.824	0.676	−1.218	0.236
X_2_	1.705	2.329	0.732	0.472
X_3_	−1.857	0.676	−2.475	0.012
X_4_	−3.337	2.329	−1.433	0.165
X_5_	11.871	3.113	3.813	0.001
X_6_	6.057	1.761	3.439	0.002

* 95% confidence interval.

**Table 4:** ANOVA for response surface quadratic model of MgSO_4_ rejection rate.

Source	Sum of Squares	Df	Mean Square	F Value	*p*-Value (Prob > F)	Significance
Model	671.45	9	74.61	25.12	0.0002	**
X_3_	251.72	1	251.72	84.74	<0.0001	**
X_4_	66.17	1	66.17	22.28	0.0022	**
X_6_	84.18	1	84.18	28.34	0.0011	**
X_3_ X_4_	52.15	1	52.15	17.56	0.0041	**
X_3_ X_6_	25.62	1	25.62	8.62	0.0218	*
X_4_ X_6_	20.29	1	20.29	6.83	0.0347	*
X_3_^2^	274.06	1	274.06	92.26	<0.0001	**
X_4_^2^	3.710	1	3.71	1.25	0.3004	-
X_6_^2^	12.70	1	12.70	4.28	0.0774	-
Residual	20.79	7	2.97			
Lack of Fit	10.04	3	3.35	1.25	0.4039	-
Pure Error	10.75	4	2.69			
Total	692.24	16				
R^2^ = 0.97 Adj. R^2^ = 0.93						

Notes: ** extremely significant, * significant, - not significant.

**Table 5:** ANOVA for response surface quadratic model of membrane permeability.

Source	Sum of Squares	Df	Mean Square	F Value	*p*-Value (Prob > F)	Significance
Model	29.35	9	3.26	11.59	0.0020	**
X_3_	2.60	1	2.60	9.23	0.0189	*
X_4_	13.64	1	13.64	48.48	0.0002	**
X_6_	11.73	1	11.73	41.69	0.0003	**
X_3_X_4_	0.59	1	0.59	2.09	0.1915	-
X_3_X_6_	0.22	1	0.22	0.80	0.4019	-
X_4_X_6_	2.66	1	2.66	9.47	0.0179	*
X_3_^2^	4.20	1	4.20	14.92	0.0062	**
X_4_^2^	0.21	1	0.21	0.74	0.4167	-
X_6_^2^	2.04	1	2.04	7.24	0.0311	*
Residual	1.97	7	0.28			
Lack of Fit	1.41	3	0.47	3.36	0.1363	-
Pure Error	0.56	4	0.14			
Total	31.32	16				
R^2^ = 0.94 Adj. R^2^ = 0.86						

Notes: ** extremely significant, * significant, - not significant.

## Data Availability

The data available in this study are available on request from the corresponding author.

## Supplementary Materials

The following are available online at https://www.mdpi.com/article/10.3390/membranes12040374/s1, Table S1: Regression model results of MgCl_2_ rejection rate, Table S2: Regression model results of Na_2_SO_4_ rejection rate, Table S3: Regression model results of NaCl rejection rate, Table S4: Regression model results of membrane permeability, Table S5: Results of response surface experiments.
