# Supplementary material for: Optimization of Nanofiltration Hollow Fiber Membrane Fabrication Process Based on Response Surface Method
Source: Membranes (Basel). 2022 Mar 29;12(4):374. doi: 10.3390/membranes12040374 (PMC9032820; doi:10.3390/membranes12040374)
Supplement: Supplementary file 1 [file membranes-12-00374-s001.zip › membranes-1635416-supplementary.pdf]

Supplementary Information

Optimization of Nanofiltration Hollow Fiber Membrane Fabrication Process Based on Response Surface Method

Mingshu Wang[a,b] Chang Liu[a,b*] Min Fan[a,b] Meiling Liu[a,b] SongTao Shen[a,b]

a.School of Environment and Resources, Southwest University of Science and Technonlogy, Mianyang, 621000, China

b.Southwest University of Science and Technology low cost wastewater Treatment Technology International Science and Technology Cooperation Base of Sichuan Province, Mianyang, 621000, China

List of Tables and Figures

Table S1. Regression model results of $MgCl_2$ rejection rate.

Table S2. Regression model results of Na_2SO_4 rejection rate.

Table S3. Regression model results of NaCl rejection rate.

Table S4. Regression model results of membrane permeability.

Table S5. Results of response surface experiments

Table S1. Regression model results of $MgCl_2$ rejection rate.

Predictor	Coefficient	Standard Error	t-Value	*p*-Value
X_1	2.356	1.861	1.266	0.216
X_2	10.775	6.622	1.627	0.115
X_3	0.916	1.861	0.492	0.626
X_4	13.241	6.622	2.000	0.055
X_5	24.569	8.099	3.033	0.005
X_6	3.081	5.112	0.603	0.551

Table S2. Regression model results of Na_2SO_4 rejection rate.

Predictor	Coefficient	Standard Error	t-Value	p-Value
X_1	0.623	0.976	0.638	0.528
X_2	0.774	3.472	0.223	0.825
X_3	-0.213	0.976	-0.219	0.828
X_4	-3.136	3.472	-0.903	0.374
X_5	13.814	4.247	3.253	0.003
X_6	29.135	2.680	10.870	<0.001

Table S3. Regression model results of NaCl rejection rate.

Predictor	Coefficient	Standard Error	t-Value	p-Value
X_1	1.294	0.881	1.468	0.153
X_2	3.834	3.135	1.223	0.231
X_3	-0.009	0.881	-0.010	0.992
X_4	5.296	3.135	1.690	0.102
X_5	7.204	3.834	1.879	0.070
X_6	11.283	2.420	4.663	<0.001

Table S4. Regression model results of membrane permeability.

Predictor	Coefficient	Standard Error	t-Value	*p*-Value
X_1	-0.413	0.140	-2.948	0.007
X_2	-1.505	0.482	-3.124	0.005
X_3	0.151	0.140	1.079	0.292
X_4	-1.298	0.482	-2.695	0.013
X_5	-2.425	0.644	-3.766	0.001
X_6	-3.057	0.364	-8.389	<0.001

From the results of multiple linear regression analysis:

The significant factors affecting the rejection rate of $MgCl_2$: X_5

The significant factors affecting the rejection rate of Na_2SO_4 : X_5, X_6

The significant factors affecting the rejection rate of NaCl : X_6

The significant factors affecting membrane permeability: X_1, X_2, X_4, X_5, X_6

Table S5. Results of response surface experiments.

	X_3	X_4	X_6	Membrane Permeability (LMH·MPa^{-1})	Predicted Membrane Permeability (LMH·MPa^{-1})	Relative Error(%)	$MgSO_4$ Rejection (%)	Predicted $MgSO_4$ Rejection (%)	Relative Error(%)
1	1	0	-1	12.10	12.20	0.83	69.20	69.74	0.78
2	1	1	0	11.30	10.64	5.84	80.12	81.31	1.49
3	-1	-1	0	12.00	12.27	2.25	86.85	86.28	0.35
4	0	0	0	9.60	9.75	1.56	87.38	88.24	0.97
5	-1	0	1	8.50	8.53	0.35	88.36	87.61	0.85
6	1	1	1	7.00	7.52	7.43	92.28	91.45	0.90
7	1	0	1	9.50	9.19	3.26	81.11	81.47	0.44
8	0	1	-1	9.70	10.06	3.71	89.55	88.13	1.59
9	0	0	0	10.20	9.75	0.45	89.50	88.24	1.41
10	0	-1	-1	11.40	11.01	3.42	86.43	87.05	0.71
11	0	0	0	9.90	9.75	1.52	89.00	88.24	0.85
12	0	0	0	9.60	9.75	1.56	89.82	88.24	1.76
13	0	0	0	9.20	9.75	5.98	85.92	88.24	2.63
14	-1	1	0	9.00	8.78	2.44	84.30	85.36	1.24
15	-1	0	-1	10.70	10.62	0.75	85.42	85.68	0.30
16	0	-1	1	10.50	10.26	2.29	87.70	88.91	1.36
17	1	-1	0	12.30	12.65	2.85	69.52	68.26	1.41
